# Bridging Microbiota Science and Clinical Practice in Pediatric Healthcare: A National Survey

**DOI:** 10.3390/children13081016

**Published:** 2026-07-30

**Authors:** Mariarosaria Matera, Ilaria Cavecchia, Maria Teresa Illiceto, Matilde Morandin, Maria Beatrice Lenzi, Talia D’Ambrosio, Valentina Biagioli

**Affiliations:** 1 Department of Pediatric Emergencies, Misericordia Hospital, 58100 Grosseto, Italy; 2 Microbiomic Department, Koelliker Hospital, 10134 Turin, Italy; 3 Pediatric Gastroenterology and Digestive Endoscopic Unit, Department of Pediatrics, “Santo Spirito” Hospital of Pescara, 65124 Pescara, Italy; 4 Neonatal Intensive Care Unit, Villa Sofia Cervello Hospital, 90146 Palermo, Italy; 5 Pediatric Clinic, University of Ferrara, 44121 Ferrara, Italy; 6 Primary Care Pediatrician, Ferrara Local Health Authority (AUSL Ferrara), 44121 Ferrara, Italy; 7 Department of Neuroscience, Rehabilitation, Ophthalmology, Genetics, Maternal and Child Health, University of Genoa, 16126 Genoa, Italy

**Keywords:** pediatric microbiome, gut microbiota, healthcare professionals, microbiota education, clinical practice, probiotics, perinatal care, knowledge, attitudes, practices, professional training, cross-sectional survey

## Abstract

**Highlights:**

**What are the main findings?**

- Italian healthcare professionals involved in pediatric and perinatal care reported highly positive attitudes toward the clinical relevance of the microbiota, but heterogeneous levels of self-perceived knowledge and translational preparedness.
- Previous microbiota-related training was independently associated with higher self-perceived knowledge scores and greater integration of microbiota-oriented approaches into clinical practice.

**What are the implications of the main findings?**

- The findings identify microbiota-related education as a major unmet need and support the development of structured, multidisciplinary educational pathways for healthcare professionals.
- Evidence-based educational initiatives may facilitate the responsible integration of microbiota-related knowledge, probiotic use, and microbiota-oriented clinical approaches into pediatric and perinatal healthcare.

**Abstract:**

The human microbiota is increasingly recognized as a key contributor to pediatric and perinatal health. However, little is known about healthcare professionals’ preparedness to integrate microbiota-related concepts into routine pediatric and perinatal care. **Methods**: A cross-sectional online survey was conducted among Italian healthcare professionals involved in pediatric and perinatal care. The questionnaire explored microbiota-related training, self-perceived knowledge, attitudes, clinical practices, microbiota-testing-related approaches, and educational needs. Descriptive and inferential statistical analyses were performed. **Results**: A total of 441 participants were included in the final analysis. Participants expressed highly positive attitudes toward the clinical relevance of the microbiota, particularly regarding maternal–fetal programming, neonatal development, preventive medicine, and microbiota education. Self-perceived knowledge was generally higher for basic microbiota concepts and neonatal microbiota development, whereas lower confidence emerged for microbiota-related clinical applications, counseling competencies, and critical interpretation of scientific evidence. Significant variability in microbiota-related knowledge scores emerged across professional backgrounds and healthcare settings (*p* < 0.001). Previous microbiota-related training was independently associated with higher self-perceived knowledge scores and greater integration of microbiota-oriented approaches into clinical practice. Use of probiotics was reported by 72.6% of participants, whereas microbiota-related diagnostic tests were routinely used by 32.9%. Overall, 91.8% of respondents expressed interest in further microbiota-related education. **Conclusions**: Healthcare professionals involved in pediatric and perinatal care recognize the growing clinical relevance of microbiota science but report heterogeneous levels of translational preparedness and clinical confidence. Microbiota-related training emerged as a key factor associated with greater preparedness and implementation in clinical practice, highlighting the need for structured multidisciplinary educational pathways and evidence-based integration of microbiome science into pediatric healthcare.

## 1. Introduction

The human microbiota is increasingly recognized as a key contributor to pediatric and perinatal health. The human gut microbiota is a complex and dynamic microbial ecosystem that contributes to essential physiological processes, including digestion, immune modulation, inflammation control, and protection against pathogens. In pediatrics, it is particularly relevant because microbial colonization occurs alongside rapid immune, metabolic, gastrointestinal, and neurodevelopmental maturation. The first 1000 days of life, from conception to approximately two years of age, represent a critical developmental window during which microbial communities are shaped by maternal factors, mode of delivery, gestational age, breastfeeding, complementary feeding, antibiotic exposure, infections, and environmental influences [1,2,3,4]. Breastfeeding and vaginal delivery have been associated with beneficial health outcomes, whereas early microbiota alterations have been associated with an increased risk of chronic conditions later in life [1,2].

Growing evidence suggests that early-life microbiota composition may influence immune, metabolic, gastrointestinal, and neurodevelopmental trajectories through complex host–microbe interactions [3,4,5,6,7,8,9,10], although the strength of evidence varies across clinical conditions. Accordingly, microbiota-related mechanisms have been investigated in a wide range of pediatric conditions, including necrotizing enterocolitis, allergic diseases, disorders of gut–brain interaction, celiac disease, neurodevelopmental disorders, pediatric oncology, and hematopoietic stem cell transplantation-related complications [11,12,13,14,15,16,17,18,19]. Although causality remains incompletely defined in many clinical contexts, these findings have stimulated growing interest in microbiota-oriented approaches within preventive and personalized medicine.

Interest in microbiome science has expanded rapidly in recent years, accompanied by increasing availability of microbiota testing and microbiota-informed healthcare approaches. However, substantial challenges remain regarding the evidence-based implementation of these tools in routine clinical practice. A major limitation concerns the lack of methodological and interpretative standardization across commercially available microbiota tests, with considerable variability in analytical methods, sequencing technologies, reference databases, and bioinformatic pipelines [20]. Furthermore, despite growing interest in microbiome-based biomarkers, no microbiota-derived biomarker is currently used in routine medical practice because of limited standardization, poor comparability across studies, and insufficient clinical validation [21]. Consequently, a substantial gap persists between the expanding scientific knowledge on the microbiome and its translation into evidence-based clinical care [22].

Healthcare professionals play a central role in translating microbiota-related evidence into patient counselling, preventive strategies, clinical decision-making, and family education. Nevertheless, educational exposure to microbiome science varies substantially across specialties and professional backgrounds, potentially influencing confidence, attitudes, and implementation in routine practice. Evaluating healthcare professionals’ self-perceived knowledge, attitudes, confidence, and real-world clinical practices is essential to identify educational needs and support the evidence-based integration of microbiota-related concepts into pediatric and perinatal healthcare, particularly because data in this area remain limited.

Although previous surveys have explored healthcare professionals’ perspectives on microbiota-related topics within specific pediatric subspecialties, particularly the survey by Brenig et al. conducted in pediatric gastroenterology [23], comprehensive multidisciplinary data encompassing pediatric and perinatal healthcare professionals remain limited. Therefore, this national cross-sectional survey aimed to evaluate microbiota-related self-perceived knowledge, attitudes, clinical practices, and educational needs among Italian healthcare professionals involved in pediatric and perinatal care. Specifically, we assessed healthcare professionals’ self-perceived knowledge across key microbiota domains, attitudes toward the clinical relevance of the microbiota, integration of microbiota-oriented approaches into routine care, factors associated with greater perceived preparedness, and unmet educational needs to support future educational initiatives in this rapidly evolving field.

## 2. Materials and Methods

### 2.1. Study Design

This study was designed as a cross-sectional observational survey to evaluate knowledge, attitudes, training, and clinical practices related to the microbiota among healthcare professionals, including those in training. The survey explored participants’ educational background on microbiota-related topics; self-perceived knowledge across key microbiota domains; perceived clinical relevance of the microbiota in pediatric health and disease; microbiota-oriented clinical practices; microbiota-testing-related practices; and interest in further education and research in this field.

The study population included healthcare professionals working in different clinical and educational settings, including hospital-based, university-based, territorial, and mixed healthcare environments. Participants represented several clinical specialties, with a particular focus on maternal and child healthcare areas.

**Figure 1:**
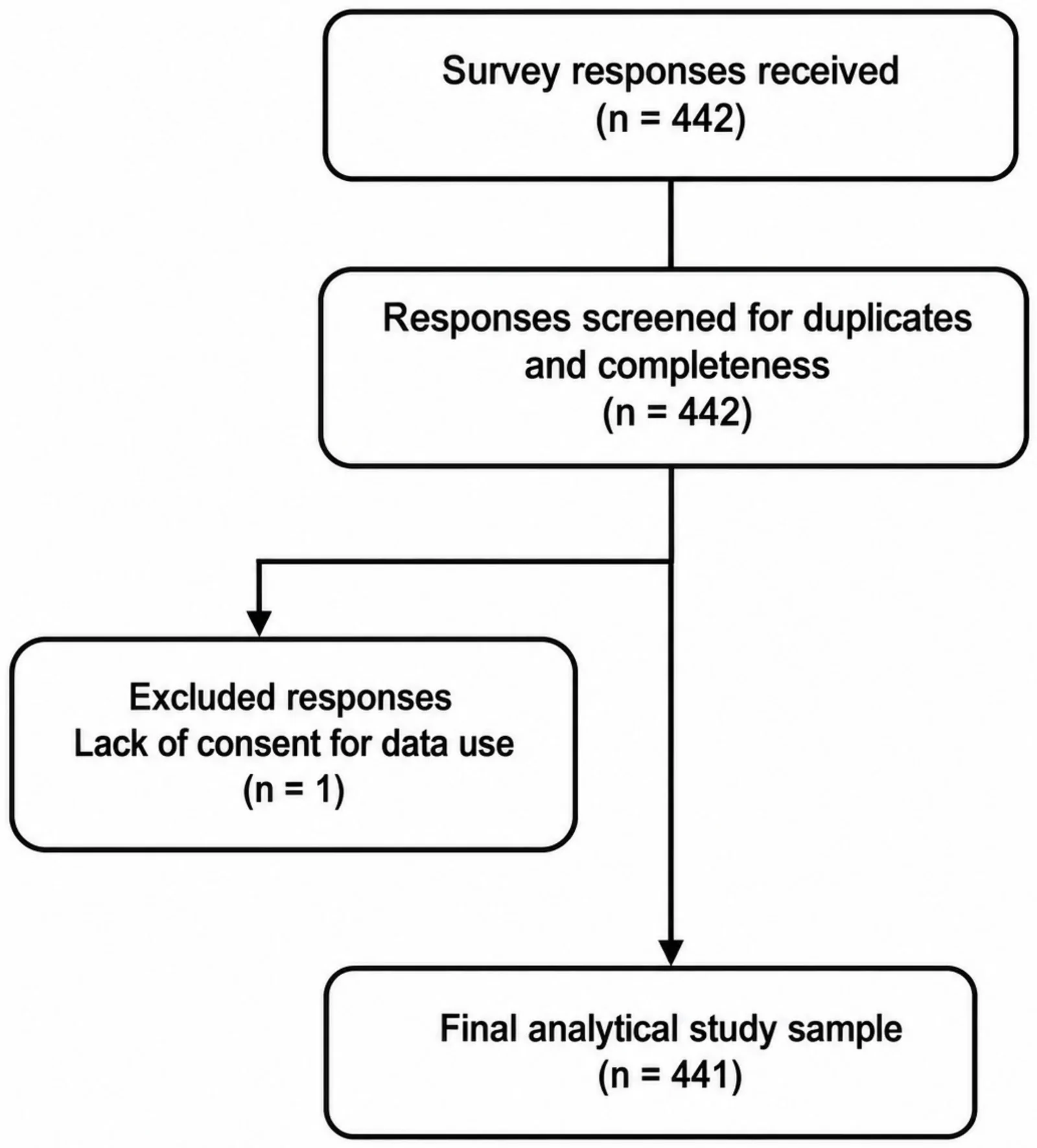
Participant flowchart and dataset selection process.

Data were collected anonymously through a structured questionnaire administered online. Participation was voluntary, and all respondents provided informed consent before completing the survey. No personally identifiable information was collected. Responses were exported into a dedicated database, subsequently cleaned and standardized, and prepared for statistical analysis (Figure 1).

### 2.2. Participants

The survey targeted Italian healthcare professionals involved in pediatric and perinatal care, including pediatricians, neonatologists, pediatric subspecialists, midwives, nurses, dentists, nutrition specialists, healthcare professionals in training, and other healthcare professionals involved in pediatric care.

Participants were recruited nationwide through a web-based dissemination strategy. The survey link was distributed via professional mailing lists, scientific societies, multidisciplinary networks, and healthcare professional associations. Several Italian scientific societies and professional networks involved in pediatric, neonatal, nutritional, neuropsychiatric, gastroenterological, respiratory, and perinatal care, as well as dental healthcare, were contacted and invited to support the dissemination of the survey through institutional mailing lists, newsletters, websites, and social media channels.

Additional dissemination occurred during scientific meetings, congresses, and professional educational events in the fields of pediatrics and neonatology and related maternal–child healthcare disciplines.

Participation was voluntary and anonymous. Eligibility criteria included being a healthcare professional involved in pediatric or perinatal clinical practice or training in Italy. The survey was administered using Google Forms (Google LLC, Mountain View, CA, USA), with all questionnaire items set as mandatory. Consequently, only fully completed questionnaires could be submitted and were therefore included in the final analysis.

### 2.3. Questionnaire Development

The questionnaire was developed through a multidisciplinary expert-based process within the Neonatal and Pediatric Clinical Microbiomics Study Group (GdS NeoPed MICS), a national multidisciplinary study group composed of 21 healthcare professionals with expertise in pediatric and perinatal microbiota-related care. The development panel included neonatologists, general pediatricians, primary care pediatricians, pediatric gastroenterologists, pediatric pulmonologists, pediatric surgeons, infectious disease specialists, obstetricians and midwives, nurses, biologists, nutritionists, dietitians, and pediatric dentists.

Survey items were generated following a literature review and multidisciplinary group discussions focused on microbiota-related knowledge, perceived clinical relevance, microbiota-oriented clinical practices, microbiota-testing-related approaches, and educational needs in pediatric and perinatal care.

Before dissemination, the questionnaire underwent internal pilot testing within the multidisciplinary study group to assess item relevance, clarity, comprehensibility, consistency of interpretation, and survey completion time. Minor revisions were implemented before final administration.

The final questionnaire included items exploring professional background, previous microbiota-related training, self-perceived knowledge across key microbiota domains, perceived relevance of host–microbiota interactions in pediatric diseases, microbiota-oriented clinical practices, microbiota-testing-related practices, and educational interests. The questionnaire was specifically developed for this national cross-sectional survey and was intended as an exploratory research instrument rather than a standardized psychometric tool. Accordingly, although it underwent multidisciplinary expert review and internal pilot testing to improve content relevance, clarity, comprehensibility, and feasibility, formal psychometric validation (including assessment of construct validity, internal consistency, criterion validity, and test–retest reliability) was not performed. Therefore, the findings should be interpreted in light of the exploratory nature of the instrument. The complete original Italian questionnaire administered to participants is provided as Supplementary File S1 to ensure transparency and allow evaluation of the survey instrument used in this study.

### 2.4. Questionnaire Domains

The questionnaire was organized into thematic domains aimed at investigating participants’ educational background, theoretical knowledge, clinical attitudes, and professional practices related to the microbiota.

Professional and occupational characteristics

The Section 1 collected demographic and professional information, including healthcare setting, clinical specialty, professional role, years of clinical experience, and geographical area of professional activity.

Educational background and training

This domain explored participants’ previous exposure to microbiota-related education, including formal academic courses, professional training programs, conferences, scientific literature, and self-directed learning activities. Participants were specifically asked whether they had previously received education on microbiota-related topics and to indicate one or more sources of training. Response options included formal university education, face-to-face professional courses, online courses, scientific congresses, self-directed learning through scientific literature, other educational experiences, or no previous education on microbiota-related topics. For statistical analyses, participants reporting at least one educational source were classified as having received previous microbiota-related education, whereas those selecting “No” were classified as having no previous microbiota-related education.

Knowledge of microbiota composition and functions

This section explored self-perceived knowledge regarding microbiota composition, physiological functions, determinants of microbiota development, mechanisms associated with dysbiosis, microbiota analysis, interpretation of microbiota-related findings, and microbiota-oriented therapeutic approaches. Questions addressed the role of nutrition, antibiotics, environmental factors, maternal and neonatal influences, and microbiota-related health outcomes.

Perceived clinical relevance of the microbiota

Participants were asked to rate the perceived relevance of host–microbiota interactions across a range of pediatric and perinatal conditions, including neonatal diseases, allergic disorders, gastrointestinal diseases, obesity, autoimmune diseases, neurodevelopmental disorders, neurological conditions, and pediatric oncology.

Clinical attitudes and microbiota-related practices

Participants were asked about the integration of microbiota-related concepts into clinical practice, including the reported use or recommendation of probiotics, prebiotics, dietary interventions, other microbiota-oriented interventions, and microbiota-testing-related practices.

Additional items explored the use of microbiota-oriented interventions for preventive and therapeutic purposes, as well as approaches to microbiota test prescription and interpretation.

Research interest and educational needs

The final domain assessed interest in additional training opportunities, scientific updates, participation in microbiota-related research activities, and involvement in professional or scientific communities focused on microbiota-related topics.

Responses from self-perceived knowledge-related items were converted into numerical values to generate composite microbiota-related knowledge scores for subsequent statistical analyses.

### 2.5. Data Collection

Data collection was performed using a structured online questionnaire specifically developed for the purposes of this study. The survey was administered through Google Forms and distributed electronically to eligible participants during the study period. Data collection was conducted between June 2025 and May 2026. During this period, the survey was disseminated through scientific societies, professional networks, educational events, and online communication channels targeting healthcare professionals involved in pediatric and perinatal care. Participation was anonymous and voluntary, and electronic informed consent was obtained from all participants before access to the questionnaire was granted. Self-perceived knowledge-related items were scored using predefined numerical scales, and a composite knowledge score was calculated. Additional derived variables were created during the data cleaning phase, including a binary indicator of previous microbiota-related education. Participants reporting at least one educational source were classified as having received previous microbiota-related education, whereas those reporting no previous education were classified accordingly. Additional binary indicators were also created for the reported use or recommendation of microbiota-related interventions (including probiotics) and for microbiota-related diagnostic testing practices.

The final cleaned dataset included 441 completed responses and all variables required for the planned statistical analyses. Data cleaning procedures included standardization of categorical variables, verification of data consistency, and creation of grouped variables for subsequent statistical analyses. Because all questionnaire items were mandatory, no missing responses were present, and no imputation or missing-data handling procedures were required.

### 2.6. Statistical Analysis

Descriptive statistics were used to summarize participant characteristics and survey responses. Categorical variables were reported as frequencies and percentages. Continuous variables were summarized as mean ± standard deviation (SD), while ordinal variables were summarized using median and interquartile range (IQR), as appropriate.

Comparisons across groups were performed using chi-square tests for categorical variables and Mann–Whitney U test or Kruskal–Wallis test for ordinal variables.

Composite microbiota-related knowledge scores were calculated by summing the numerical values assigned to nine self-perceived knowledge-related Likert-scale items included in the questionnaire. These items assessed participants’ self-perceived competence regarding the definition and functions of the human microbiota, microbiota development and factors influencing its composition, microbiota modulation strategies, microbiota analysis and clinical interpretation, knowledge of prebiotics, probiotics, synbiotics and postbiotics, and the selection of personalized microbiota-targeted therapeutic approaches. Each item was scored on a five-point Likert scale ranging from 1 (“no knowledge”) to 5 (“expert knowledge”). All items contributed equally to the composite score without weighting, yielding a theoretical composite score ranging from 9 to 45, with higher scores indicating greater self-perceived microbiota-related knowledge. Because all questionnaire items were mandatory, no missing responses occurred; therefore, no imputation or missing-data handling procedures were required. Because knowledge scores were derived from ordinal Likert-scale responses, non-parametric statistical methods were used for group comparisons. To further explore the independent association between previous microbiota-related training and self-perceived microbiota-related knowledge, a multivariable linear regression analysis was performed using the composite knowledge score as the dependent variable. Previous microbiota-related training, professional macro-category, healthcare setting, and years of professional experience were included as independent variables.

The complete questionnaire is available as Supplementary Material File S1.

### 2.7. Ethics Statement

Participation in the survey was voluntary and anonymous. Electronic informed consent was obtained from all participants before questionnaire completion. No identifiable personal, sensitive, or health-related data were collected. The survey was administered exclusively to healthcare professionals and did not involve patients or biological samples. Therefore, ethics committee approval was not sought. The study was conducted in accordance with the ethical principles of the Declaration of Helsinki and applicable Italian data protection legislation.

## 3. Results

### 3.1. Participant Characteristics: Classification of Professional Groups

Participants (*n* = 441) were classified according to their professional background using a predefined hierarchical categorization system. Individual professional roles were grouped into broader clinically meaningful macro-categories to facilitate data interpretation and presentation. Categories were mutually exclusive and defined according to clinical function and domain of care.

Absolute frequencies (*n*) and relative percentages (%) were calculated using the total sample size as the denominator. Participant distribution according to professional macro-categories and clinical specialties is summarized in Table 1, whereas professional setting, years of experience, and geographical distribution stratified by macro-category are reported in Table 2.

Unless otherwise specified, descriptive and inferential analyses were performed using these predefined professional macro-categories. Analyses of clinical practices and educational needs (Section 3.5 and Section 3.6) were instead performed at the level of individual professional groups (i.e., the clinical specialties listed in Table 1) to better capture profession-specific differences in routine clinical practice and educational priorities.

The study population was predominantly composed of professionals working in maternal and perinatal care (44.44%) and neonatology/general pediatrics (38.55%), together accounting for more than 80% of the study sample. Within the maternal and perinatal care category, the vast majority of participants were midwives or midwifery students (194/196; 99.0%). Smaller proportions of participants represented pediatric medical specialties (2.95%), surgical specialties (0.68%), oral and dental care (4.08%), neurodevelopmental and rehabilitation sciences (4.31%), nursing and allied pediatric care (0.23%), and nutrition and laboratory sciences (4.76%).

As shown in Table 2, professionals involved in maternal and perinatal care were distributed across both hospital-based and territorial/community settings, whereas participants belonging to the neonatology and general pediatrics categories were predominantly based in territorial/community settings. Across the overall study population, most respondents were experienced professionals, with more than 20 years of professional activity, and Northern Italy represented the most frequent geographical area of practice.

### 3.2. Self-Perceived Knowledge Levels

Participants’ self-perceived knowledge regarding microbiota-related topics was assessed through multiple Likert-scale items exploring general microbiota knowledge, maternal–fetal microbial transmission, neonatal microbiota development, clinical applications, and confidence in counseling practices.

Likert-scale responses were treated as ordinal variables and summarized using median and interquartile range (IQR). Distribution of self-perceived microbiota-related knowledge across clinical domains is summarized in Table 3.

Overall, participants reported moderate-to-high self-perceived knowledge levels regarding general microbiota concepts and neonatal microbiota development. Lower confidence scores were observed for microbiota-related clinical applications, counseling competencies, and critical interpretation of scientific evidence, suggesting a gap between theoretical awareness and perceived translational preparedness.

### 3.3. Attitudes/Perceptions

Participants’ attitudes and perceptions regarding the role of the microbiota in maternal, neonatal, and pediatric health were assessed through Likert-scale items exploring perceived clinical relevance, impact on neurodevelopment, preventive potential, adequacy of current scientific evidence, and future integration into clinical practice.

**Figure 2:**
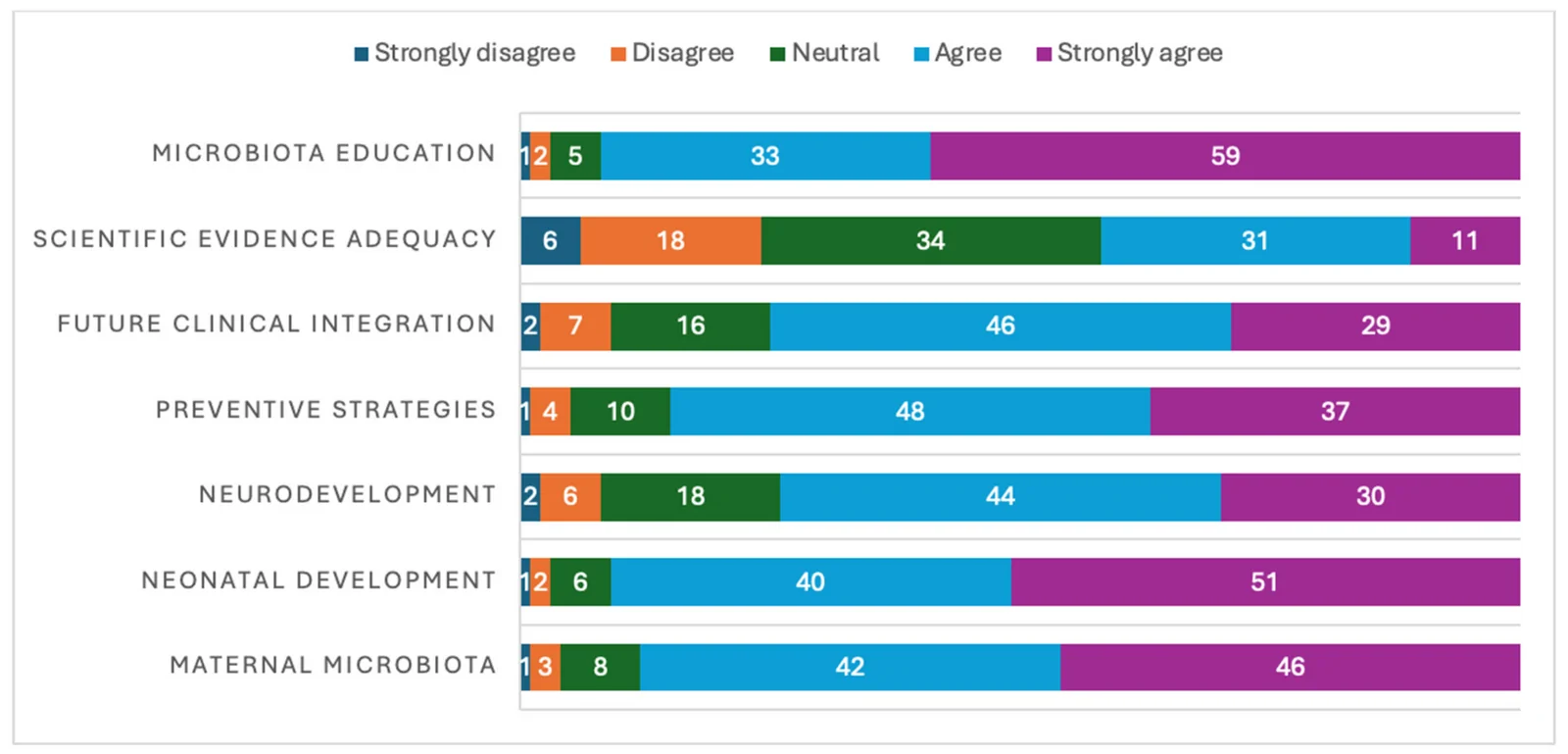
Distribution of Likert-scale responses across attitudes/perceptions domains.

Likert-scale responses were analyzed as ordinal variables and summarized using median and interquartile range (IQR). Distribution of responses across perception domains is reported in Table 4 and graphically represented in Figure 2.

**Figure 3:**
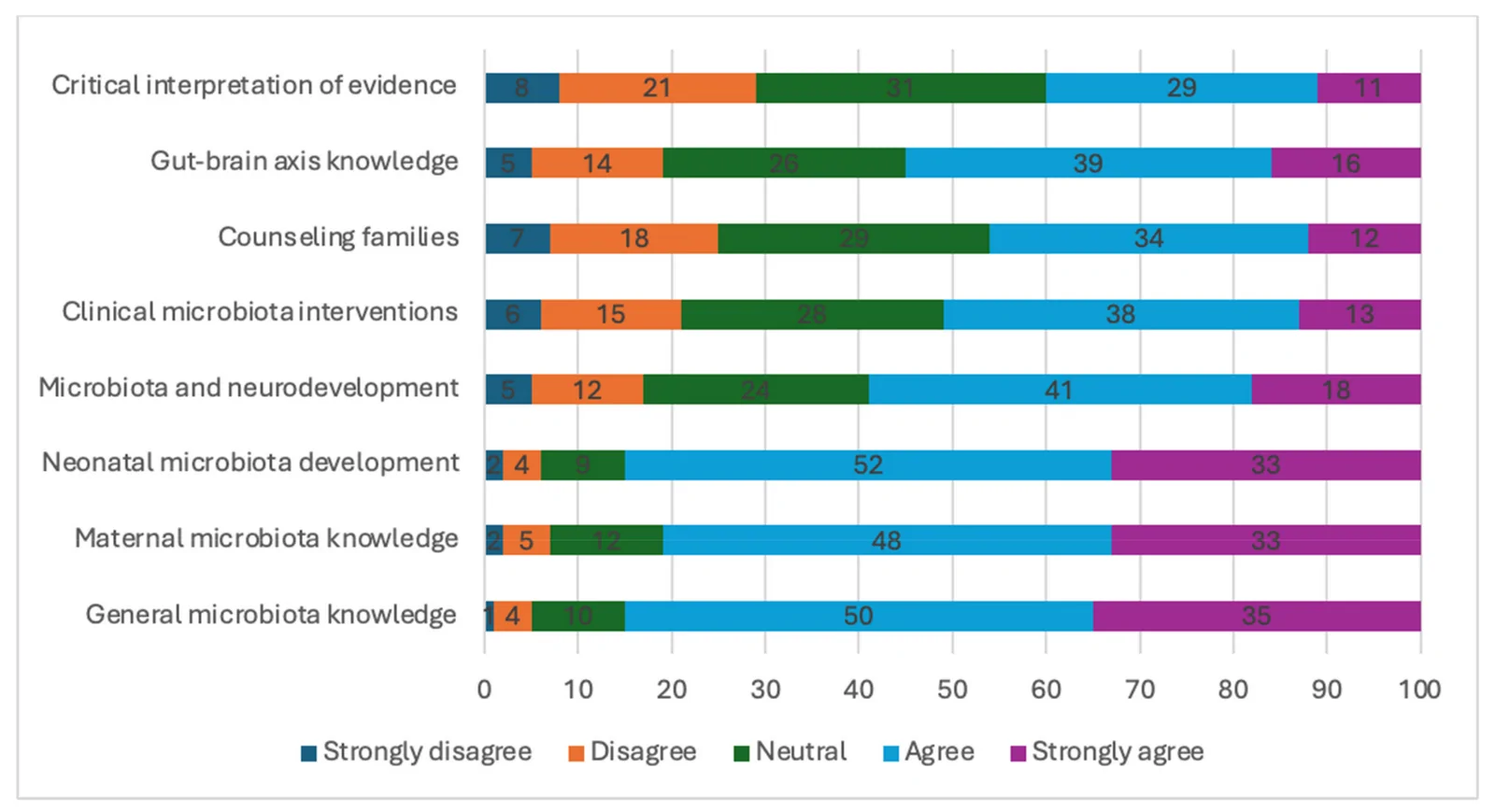
Distribution of Likert-scale responses across self-perceived microbiota-related knowledge domains.

Overall, participants expressed highly positive attitudes toward the clinical relevance of the microbiota. The highest levels of agreement were observed for the importance of the maternal microbiota during pregnancy, the role of the microbiota in neonatal development, and the relevance of microbiota education in healthcare training (all median = 5, IQR 4–5). Positive perceptions were also reported regarding microbiota-oriented preventive strategies and the future clinical integration of microbiota-based interventions (median = 4). In contrast, perceptions regarding the adequacy of the current scientific evidence were more heterogeneous and received the lowest agreement scores (median = 3, IQR 2–4), suggesting greater uncertainty about the strength and clinical applicability of the available evidence. The distribution of self-perceived microbiota-related knowledge levels across the assessed domains is shown in Figure 3.

Stacked horizontal bar plots illustrate the percentage distribution of self-perceived knowledge levels across microbiota-related domains. Higher levels of self-perceived knowledge were reported for general microbiota concepts, maternal microbiota, and neonatal microbiota development. Lower confidence levels were observed for microbiota-related clinical interventions, counseling competencies, gut–brain axis knowledge, and critical interpretation of microbiota-related scientific evidence, highlighting a gap between theoretical awareness and perceived translational preparedness.

### 3.4. Inferential Analyses

Inferential analyses revealed significant differences in self-perceived microbiota-related knowledge scores according to professional background, healthcare setting, and previous microbiota-related training (Table 5).

Significant differences in self-perceived microbiota-related knowledge scores were observed across professional macro-categories (Kruskal–Wallis, *p* < 0.001), indicating heterogeneous levels of self-perceived microbiota-related knowledge among the surveyed professional groups.

Significant differences also emerged according to professional setting. Territorial/community professionals reported slightly higher knowledge scores compared with hospital-based professionals (median 23 [IQR 18–29] vs. 20 [16–27], Mann–Whitney U *p* = 0.004).

Participants reporting previous microbiota-related training demonstrated substantially higher self-perceived knowledge scores compared with those without specific training (median 24 [18–31] vs. 13 [11–18], Mann–Whitney U *p* < 0.001). To determine whether this association remained after adjustment for potential confounding factors, a multivariable linear regression analysis was performed (Table 6). The model included previous microbiota-related training, professional macro-category, healthcare setting, and years of professional experience as independent variables. After adjustment, previous microbiota-related training remained independently associated with higher knowledge scores (β = 8.35, 95% CI 6.59–10.11; *p* < 0.001). Years of professional experience were also independently associated with higher knowledge scores (β = 1.08, 95% CI 0.45–1.71; *p* = 0.001). Compared with the reference category (Maternal and perinatal care), participants from Neonatology and general pediatrics (β = 2.69, 95% CI 1.14–4.25; *p* = 0.001), Pediatric medical specialties (β = 4.52, 95% CI 0.46–8.58; *p* = 0.029), and Nutrition and laboratory sciences (β = 11.13, 95% CI 7.85–14.40; *p* < 0.001) showed significantly higher knowledge scores.

### 3.5. Clinical Practices

The clinical practices domain investigated the integration of microbiota-related approaches into routine clinical activity. In particular, participants were asked about their reported use or recommendation of probiotics and whether they used microbiota-related diagnostic tests in clinical practice. These variables were analyzed as categorical binary variables (yes/no).

Descriptive analyses were performed using absolute frequencies and percentages. Among the study population, 320 participants (72.6%) reported using or recommending probiotics in routine clinical practice, whereas 121 participants (27.4%) did not report using or recommending probiotics. Microbiota-related diagnostic tests were used by 145 participants (32.9%), while 296 participants (67.1%) reported not using such tests in routine practice.

Associations between categorical variables were evaluated using chi-square tests. In particular, the relationship between previous microbiota-related training and microbiota-oriented clinical practices was explored.

**Figure 4:**
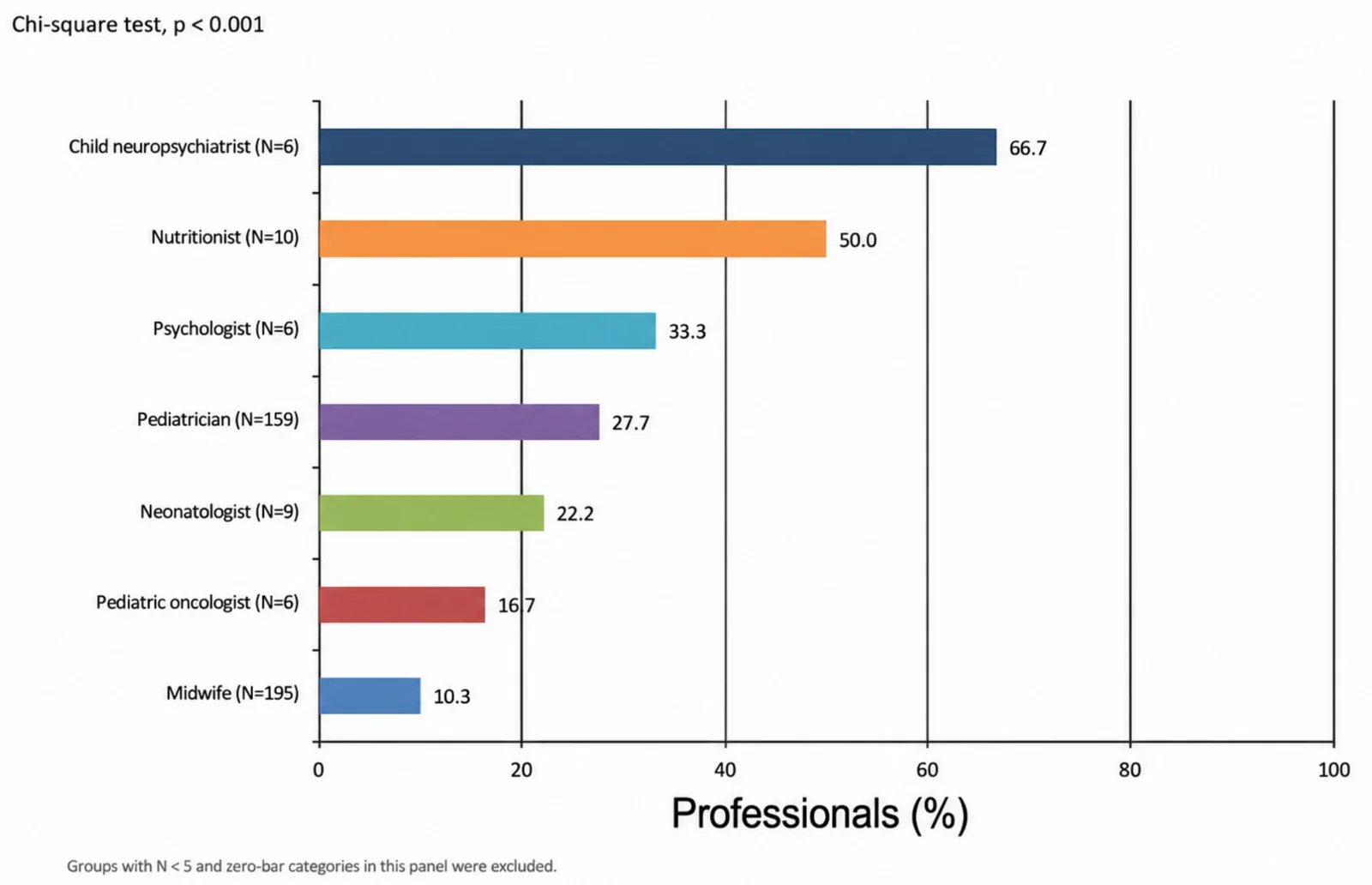
Reported use of probiotics across professional groups. Percentage of participants within each professional group reporting the use or recommendation of probiotics (“Yes”) in routine clinical practice according to professional background. Statistical significance was assessed using the chi-square test (*p* < 0.001).

**Figure 5:**
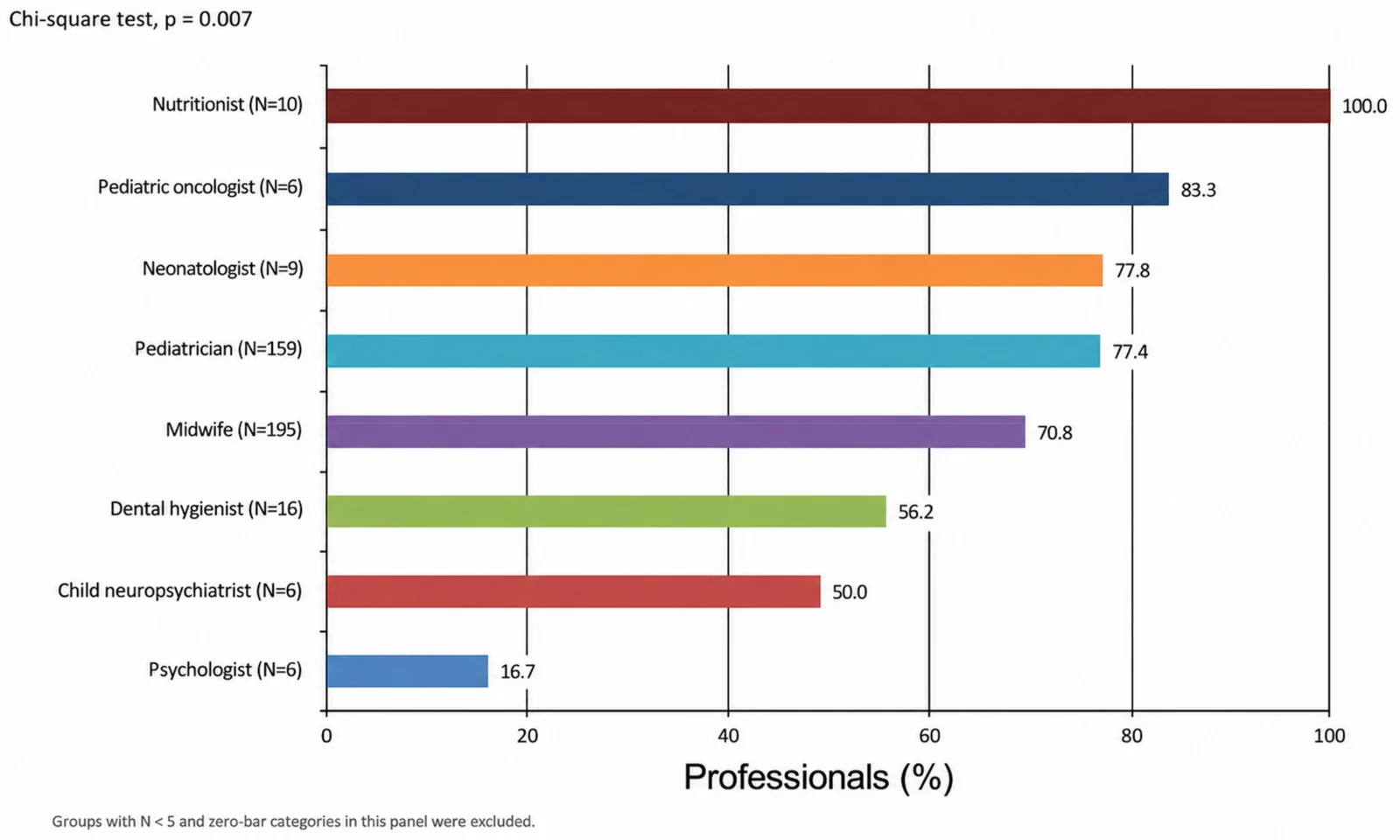
Use of microbiota-related diagnostic tests across professional groups. Percentage of participants within each professional group who reported the use of microbiota-related diagnostic tests (“Yes”) in routine clinical practice. Groups with fewer than five participants and categories with no reported test use were excluded from the analysis. Statistical significance was assessed using the chi-square test (*p* = 0.007).

Participants with previous microbiota-related training showed significantly higher rates of microbiota-oriented clinical practice compared with participants without previous training (χ^2^ = 12.55, *p* = 0.0004). Conversely, the association between microbiota-related training and prescription of microbiota-related diagnostic tests did not reach conventional statistical significance (χ^2^ = 3.77, *p* = 0.052), although a positive trend was observed. The percentages reported above refer to the overall study population (*n* = 441), whereas Figure 4 and Figure 5 present the corresponding proportions within each professional group. Significant differences in the reported use of probiotics were observed across professional groups (χ^2^ test, *p* < 0.001; Figure 4). Among the smaller professional subgroups, 4 of 6 child neuropsychiatrists (66.7%) and 5 of 10 nutritionists (50.0%) reported using or recommending probiotics. Lower proportions were observed among neonatologists (22.2%), pediatric oncologists (16.7%), and midwives (10.3%). Pediatricians and psychologists showed intermediate proportions of reported probiotic use (27.7% and 33.3%, respectively). Because several professional categories included only a limited number of participants, these subgroup-specific estimates should be interpreted with caution. Detailed subgroup-specific percentages are reported in Figure 4. Overall, previous microbiota-related training was associated with higher rates of microbiota-oriented clinical practice. In addition, the reported use of probiotics was substantially more common than the use of microbiota-related diagnostic testing across the study population.

### 3.6. Educational Needs

**Figure 6:**
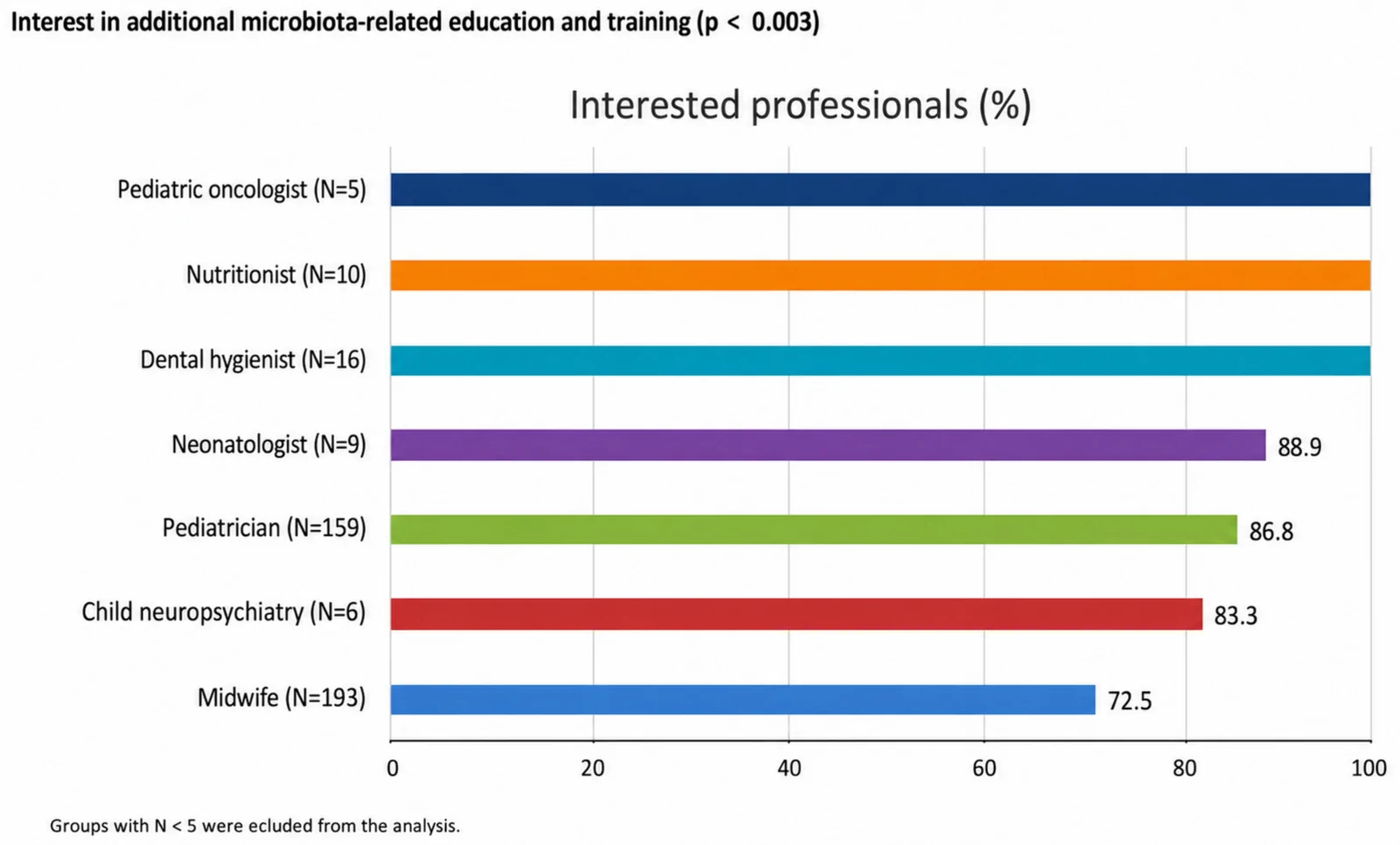
Educational needs across professional groups. Horizontal bar plot showing the percentage of participants expressing interest in additional microbiota-related education and training according to professional background. Groups with fewer than five participants were excluded from the analysis. Significant differences were observed across professional groups (χ^2^ test, *p* < 0.003).

Participants were asked about their interest in further microbiota-related education and the educational topics they considered most relevant for future professional development. Responses were analyzed at the level of individual professional groups to identify profession-specific educational priorities. Descriptive data are presented as absolute frequencies and percentages, and differences between professional groups were evaluated using the chi-square test. Overall, the analysis demonstrated a remarkably high level of interest in further microbiota-related education among healthcare professionals included in the survey. Overall, 91.8% of respondents (405/441) reported interest in deepening their knowledge regarding the relationship between the microbiota and pediatric health, while only 2.0% (9/441) reported no interest and 6.1% (27/441) expressed uncertainty. Interest in additional microbiota-related education was consistently observed across all professional groups, although significant variability emerged between specialties (χ^2^ test, *p* < 0.003; Figure 6).

Among the surveyed professional groups, high levels of interest in further microbiota-related education were observed across all categories, with percentages ranging from 72.5% to 100. However, because several professional groups included only a limited number of participants, subgroup-specific percentages should be interpreted with caution and considered exploratory rather than representative of the corresponding professional populations. The educational topics most frequently requested by participants included microbiota modulation strategies, the clinical use of probiotics, prebiotics, synbiotics, and postbiotics, interpretation of fecal microbiota analyses, microbiota-related preventive medicine, the gut–brain axis and neurodevelopmental disorders, as well as gastrointestinal and immune-mediated diseases. Furthermore, a substantial proportion of participants expressed interest in participating in multidisciplinary study groups, clinical communities of practice, and collaborative research initiatives focused on microbiota science.

Overall, microbiota-related education emerged as a major area of interest across pediatric and allied healthcare specialties.

## 4. Discussion

### 4.1. Main Findings

The present study shows that Italian healthcare professionals involved in pediatric and perinatal care broadly recognize the clinical relevance of the microbiota, yet their translational preparedness remains heterogeneous, particularly in areas such as counseling, clinical application, and critical appraisal of the evidence.

This pattern is consistent with previous observations in pediatric gastroenterology, where interest in microbiome analysis and fecal microbiota transfer is high, but implementation is constrained by limited standardization and the need for clearer clinical guidance [23]. Although this survey included a large national sample of healthcare professionals involved in pediatric and perinatal care, the distribution of professional categories reflected spontaneous participation rather than a predefined sampling strategy. Consequently, the findings primarily represent the perspectives of professionals working in maternal–perinatal care and general pediatrics, while subgroup analyses involving less represented professional categories should be interpreted cautiously as exploratory rather than representative of the entire Italian pediatric healthcare workforce.

### 4.2. Knowledge Gap

A key finding is that previous microbiota-related training remained independently associated with higher self-perceived knowledge scores after adjustment for professional macro-category, healthcare setting, and years of professional experience. In addition, participants reporting previous microbiota-related training showed greater integration of microbiota-oriented approaches into clinical practice. However, given the cross-sectional design of the study, no causal relationship can be inferred. Reverse causality and self-selection cannot be excluded, as healthcare professionals with a pre-existing interest in microbiota-related topics may have been more likely to seek additional training. Moreover, although the multivariable analysis adjusted for major professional characteristics, residual confounding related to professional background, clinical interests, or workplace characteristics may still have influenced the observed associations. Interestingly, participants working in territorial/community healthcare settings reported slightly higher self-perceived knowledge scores than those working in hospital-based settings in the univariate analysis. A possible explanation is that community-based professionals are more frequently involved in preventive care, family counselling, nutrition-related issues, and longitudinal patient management, all of which may increase exposure to microbiota-related topics. However, this difference should be interpreted cautiously, as it was modest and did not remain significant after adjustment for other professional characteristics.

Although previous microbiota-related education was associated with greater perceived preparedness, this finding should not be interpreted as evidence of a causal effect.

Because knowledge was assessed through self-perception rather than objective testing, these findings should be interpreted in light of potential self-assessment bias. Participants with previous microbiota-related education may have reported greater confidence in their competencies rather than superior objective knowledge. Nevertheless, perceived competence remains clinically relevant, as confidence may influence clinicians’ willingness to discuss microbiota-related topics with patients and families and to integrate microbiota-oriented approaches into routine clinical practice.

These findings are particularly relevant in the context of microbiota science, where rapid growth of scientific publications is often accompanied by heterogeneous dissemination of evidence into clinical practice. Structured educational initiatives may help bridge the gap between scientific advances and responsible clinical implementation by promoting the appropriate interpretation and application of microbiota-related evidence [24,25,26].

The high reported use or recommendation of probiotics observed in the survey deserves cautious interpretation. Although this high prescription rate may reflect increasing awareness of microbiota-related interventions, it may also suggest that probiotics are sometimes prescribed beyond current evidence-based, strain-specific, and condition-specific recommendations. Evidence-based recommendations in pediatric practice indicate that probiotic efficacy is strain-specific and condition-specific, and that benefits cannot be generalized across all products or indications. For example, systematic reviews of childhood functional gastrointestinal disorders have shown benefit in some abdominal pain-related conditions, but not in functional constipation. Conversely, for several other pediatric indications, current evidence remains insufficient to support the routine use of probiotics outside specific clinical contexts.

Similarly, pediatric reviews emphasize that clinicians need a solid understanding of microbiota-related concepts to avoid overinterpretation of preliminary findings and inappropriate therapeutic use [24,25,26].

In the Italian context, this evidence-based approach is further supported by national guidance issued by the Italian Ministry of Health. The *Guidelines on Probiotics and Prebiotics* establish quality and safety requirements for probiotic microorganisms marketed as food supplements, including strain identification, minimum viable counts, and appropriate labeling. Furthermore, the national guidance document *Human Microbiota: From Research to Clinical Applications* highlights the need for methodological standardization and supports the appropriate clinical interpretation and implementation of microbiota-related approaches. Together with international guidelines, these national documents provide the regulatory framework for the safe and appropriate use of probiotics and support a cautious, evidence-based, and standardized approach to reported use of probiotics and microbiota-oriented clinical practice [27,28]. This approach is also consistent with current international pediatric recommendations, which emphasize that probiotic use should remain strain-specific, indication-specific, and supported by high-quality clinical evidence.

### 4.3. Clinical Translation

The interest in microbiota-related diagnostic tests should also be interpreted in light of the current evidence base. The survey from German-speaking countries on microbiome analysis and fecal microbiota transfer highlighted that standardized procedures, clinical governance, and stronger evidence are needed before these tools can be broadly adopted in pediatric gastroenterology. In this context, the present findings suggest that growing clinical interest in microbiota-related approaches coexists with important uncertainties regarding the interpretation of current evidence and the standardization of clinical applications [9,10,11,12,13,14,15,16,17,18,19,20,21,22,23].

This discrepancy highlights the importance of developing evidence-based frameworks capable of guiding clinicians in the interpretation of microbiota-related tests and interventions. In the absence of standardized recommendations, variability in clinical practice may increase, potentially exposing patients to interventions with uncertain benefit and generating unrealistic expectations among families [29].

### 4.4. Neurodevelopment

The strong attention to neurodevelopment and the gut–brain axis is particularly noteworthy. Early-life microbiota composition has been associated with immune maturation, metabolic programming, and neurodevelopmental trajectories, although many clinical applications remain investigational rather than established. This supports the need for balanced communication: clinicians should be aware of the biological plausibility and emerging data, while avoiding overstatement of benefits that are not yet firmly supported by robust pediatric trials [30]. Although the gut–brain axis represents one of the most promising areas of microbiome research, current evidence remains largely derived from preclinical studies and observational human cohorts. Future longitudinal and interventional studies will be essential to clarify causality, identify critical developmental windows, and determine whether microbiota-targeted interventions can meaningfully influence neurodevelopmental outcomes.

### 4.5. Educational Implications

The results strongly support the need for structured, multidisciplinary, and continuing educational pathways on microbiota-related topics. The high proportion of respondents interested in further training indicates a real and transversal educational demand across professional groups. Educational programs should address basic microbiota biology, critical interpretation of evidence, appropriate use of probiotics, family counseling, and the limits of microbiota-based testing.

Importantly, education should aim not only to increase theoretical knowledge but also to strengthen translational competence. Clinicians need to understand how to contextualize evidence by age, condition, strain, dose, and clinical goal. Without this level of specificity, microbiota-related interventions risk being adopted inconsistently or prematurely.

Professional education should also foster competencies in communicating uncertainty, enabling healthcare providers to discuss microbiota-related topics with families in a scientifically accurate and ethically responsible manner. This aspect is particularly relevant in a field characterized by intense media exposure and substantial commercial interest.

The independent association between previous microbiota-related training and higher self-perceived knowledge scores suggests that targeted education initiatives may represent a valuable strategy for future professional development.

### 4.6. Strengths and Limitations

The study’s strengths include its relatively large sample size, multidisciplinary design, and real-world perspective, which provide a broad overview of microbiota-related knowledge and practices among Italian healthcare professionals. The inclusion of professionals from several healthcare disciplines provided the opportunity to explore differences across professional groups and healthcare settings, although comparisons involving smaller professional subgroups should be interpreted with caution because of their limited sample size. In addition, the questionnaire was developed through a multidisciplinary expert-based process within the Neonatal and Pediatric Clinical Microbiomics Study Group (NeoPed MICS), involving 21 healthcare professionals from different disciplines involved in pediatric and perinatal care.

Several limitations should be acknowledged. First, participation was voluntary and recruitment relied on professional societies, scientific networks, and healthcare organizations rather than probability-based sampling. Consequently, the study population reflects spontaneous participation and may overrepresent healthcare professionals with a greater interest in microbiota-related topics. Moreover, the distribution of professional categories was uneven, with maternal–perinatal care and general pediatrics accounting for most respondents, whereas several professional subgroups included only a limited number of participants (*n* ≤ 9). Therefore, subgroup-specific analyses and percentages derived from these smaller categories should be interpreted cautiously, as they may be disproportionately influenced by the responses of only a few individuals. Accordingly, these findings should be regarded as exploratory and hypothesis-generating rather than representative estimates of the corresponding professional populations or of the entire Italian pediatric healthcare workforce.

Future studies using probability-based or stratified sampling strategies and ensuring a more balanced representation of healthcare professions are warranted to confirm and extend these findings.

In addition, the questionnaire relied on self-perceived (self-reported) knowledge rather than objective testing, so the findings reflect perceived rather than measured competence. Furthermore, although the questionnaire underwent multidisciplinary expert review and internal pilot testing before dissemination it did not undergo formal psychometric validation, including assessment of construct validity, internal consistency, criterion validity, or test–retest reliability. Therefore, the questionnaire should be considered an exploratory research instrument, and this should be taken into account when interpreting the study findings.

Finally, the survey was designed to explore attitudes and practice patterns, and therefore cannot establish causal relationships between previous microbiota-related training and self-reported knowledge or clinical practice.

Furthermore, because the survey was conducted within the Italian healthcare context, caution should be exercised when extrapolating the findings to other countries characterized by different healthcare systems, educational pathways, and regulatory frameworks regarding microbiota-related interventions.

## 5. Conclusions

Overall, this study demonstrates that microbiota science has gained substantial visibility among healthcare professionals involved in pediatric and perinatal care, generating considerable interest and influencing clinical decision-making. However, the findings also reveal a persistent gap between growing scientific enthusiasm and the availability of standardized, evidence-based clinical pathways. Bridging this gap will require structured educational programs, multidisciplinary training opportunities, stronger interdisciplinary collaboration, and the development of clear clinical guidance capable of supporting the responsible integration of microbiota-related knowledge into practice.

Particular attention should be devoted to the appropriate use of probiotics, the interpretation of microbiota-based testing, and the communication of emerging evidence regarding the gut–brain axis. The independent association observed between previous microbiota-related training and higher self-perceived knowledge scores suggests that education may play a key role in improving perceived preparedness and confidence in addressing microbiota-related topics among healthcare professionals. Importantly, the present findings reflect self-perceived knowledge rather than objectively assessed knowledge, and should therefore be interpreted as indicators of perceived competence rather than actual expertise.

As microbiome research continues to evolve, healthcare professionals will increasingly be called upon to distinguish validated clinical applications from promising but still investigational hypotheses. Strengthening microbiota literacy among clinicians, while competency-based educational initiatives supported by objective assessment, may therefore represent a key prerequisite for ensuring that future advances in microbiome science translate into safe, evidence-based, and patient-centered pediatric care.

## Figures and Tables

**Table 1:** Distribution of participants according to professional macro-categories and clinical specialties (*n* = 441). Professional roles were grouped into predefined, clinically meaningful macro-categories according to primary clinical function and domain of care. Categories were mutually exclusive, and percentages were calculated using the total study population (*n* = 441) as the denominator.

Macro-Category	Clinical Specialty	*n*	%
**Maternal and perinatal care**		**196**	**44.44**
	Midwives and midwifery students	194	43.99
	Obstetrics and Gynecology	2	0.45
**Neonatology and general pediatrics**		**170**	**38.55**
	General Pediatrics and primary/emergency care	161	36.51
	Neonatology	9	2.04
**Pediatric medical specialties**		**13**	**2.95**
	Pediatric Hematology and Oncology	8	1.81
	Pediatric Endocrinology	2	0.45
	Pediatric Rheumatology	1	0.23
	Pediatric Pulmonology	1	0.23
	Pediatric Anesthesiology	1	0.23
**Surgical specialties**		**3**	**0.68**
	Pediatric Surgery	2	0.45
	General/Breast Surgery	1	0.23
**Oral and dental care**		**18**	**4.08**
	Dental Hygienists	16	3.63
	Dentistry/Odontoiatry	2	0.45
**Neurodevelopmental and rehabilitation sciences**		**19**	**4.31**
	Child Neuropsychiatry	6	1.36
	Psychology/Psychotherapy	6	1.36
	Speech and Language Therapy	2	0.45
	Developmental rehabilitation therapies	4	0.91
	Osteopathy	1	0.23
**Nursing and allied pediatric care**		**1**	**0.23**
	Pediatric Nursing	1	0.23
**Nutrition and laboratory sciences**		**21**	**4.76**

**Table 2:** Distribution of professional setting, years of experience, and geographical area across professional macro-categories. Data are presented as absolute frequencies (*n*). A professional setting refers to the participant’s primary workplace. The geographical area was classified as Northern, Central, or Southern Italy and Islands according to the reported region of professional activity.

Macro-Category	Professional Setting				Years of Experience					Geographical Area		
	Hospital-Based	Territorial/Community	University/Academic	Training	>20 Years	11–20 Years	5–10 Years	<5 Years	Not Currently Practicing	Northern Italy	Central Italy	Southern Italy and Islands
**Maternal and perinatal care**	90	85	7	14	79	50	32	22	13	84	61	51
**Neonatology and general pediatrics**	40	117	9	4	127	17	16	9	1	71	45	54
**Pediatric medical specialties**	9	1	2	1	9	2	2	0	0	4	1	8
**Surgical specialties**	0	2	1	0	1	0	2	0	0	1	1	1
**Oral and dental care**	0	3	2	13	1	1	1	2	13	11	0	7
**Neurodevelopmental and rehabilitation sciences**	2	16	0	1	8	6	1	3	1	5	7	7
**Nursing and allied pediatric care**	1	0	0	0	0	0	1	0	0	0	0	1
**Nutrition and laboratory sciences**	3	14	1	3	2	5	7	7	0	9	2	10
**Total**	145	238	22	36	227	81	62	43	28	185	117	139

**Table 3:** Self-perceived microbiota-related knowledge across clinical domains. Responses were collected using five-point Likert-scale items and summarized as median and interquartile range (IQR). Higher scores indicate greater self-perceived knowledge. Colored squares provide a visual representation of median Likert-scale scores ranging from 1 (lower self-perceived knowledge) to 5 (higher self-perceived knowledge).

Item	Median	IQR	Visual Representation
General knowledge of the human microbiota	4	3–5	■ ■ ■ ■ ■
Knowledge of maternal microbiota during pregnancy	4	3–5	■ ■ ■ ■ ■
Knowledge of neonatal microbiota development	4	3–5	■ ■ ■ ■ ■
Knowledge of microbiota and neurodevelopment	3	2–4	■ ■ ■ ■ ■
Knowledge of microbiota-related clinical interventions	3	2–4	■ ■ ■ ■ ■
Confidence in counseling families about microbiota-related topics	3	2–4	■ ■ ■ ■ ■
Knowledge of the gut–brain axis	3	2–4	■ ■ ■ ■ ■
Perceived ability to critically interpret microbiota-related evidence	3	2–4	■ ■ ■ ■ ■

**Table 4:** Attitudes and perceptions regarding microbiota-related clinical relevance. Responses were collected using five-point Likert-scale items and summarized as median and interquartile range (IQR). Higher scores indicate stronger agreement or perceived clinical relevance. Colored squares provide a visual representation of median Likert-scale scores ranging from 1 (lower agreement/perceived relevance) to 5 (higher agreement/perceived relevance).

Item	Median	IQR	Visual Representation
Perceived importance of the maternal microbiota during pregnancy	5	4–5	■ ■ ■ ■ ■
Perceived role of the microbiota in neonatal development	5	4–5	■ ■ ■ ■ ■
Perceived role of the microbiota in neurodevelopment	4	3–5	■ ■ ■ ■ ■
Perceived usefulness of microbiota-oriented preventive strategies	4	4–5	■ ■ ■ ■ ■
Confidence in the future clinical integration of microbiota-based interventions	4	3–5	■ ■ ■ ■ ■
Perceived adequacy of current scientific evidence	3	2–4	■ ■ ■ ■ ■
Perceived importance of microbiota education in healthcare training	5	4–5	■ ■ ■ ■ ■

**Table 5:** Inferential analyses of microbiota-related knowledge scores. Knowledge scores were derived from the sum of Likert-scale items exploring microbiota-related knowledge domains. Non-parametric statistical analyses were applied due to the ordinal nature of the variables.

Comparison	Groups Compared	Median Score (IQR)	Statistical Analysis	*p*-Value
Professional macro-categories	Across professional groups	Significant variability across groups	Kruskal–Wallis	<0.001
Professional setting	Hospital-based vs. territorial/community	20 (16–27) vs. 23 (18–29)	Mann–Whitney U	0.004
Previous microbiota-related training	Yes vs. No	24 (18–31) vs. 13 (11–18)	Mann–Whitney U	<0.001

**Table 6:** Multivariable linear regression analysis of factors independently associated with composite microbiota-related knowledge scores. The model was adjusted for previous microbiota-related training, professional macro-category, healthcare setting, and years of professional experience.

Variable	Adjusted β (95% CI)	*p*-Value
Previous microbiota-related training	8.35 (6.59–10.11)	<0.001
Years of professional experience	1.08 (0.45–1.71)	0.001
Neonatology and general pediatrics *	2.69 (1.14–4.25)	0.001
Pediatric medical specialties *	4.52 (0.46–8.58)	0.029
Nutrition and laboratory sciences *	11.13 (7.85–14.40)	<0.001
Mixed/other healthcare setting †	2.45 (0.02–4.88)	0.048

* Reference category: Maternal and perinatal care. † Reference category: Hospital-based setting.

## Data Availability

The data presented in this study are available on request from the corresponding author. The data are not publicly available due to privacy and ethical reasons.

## Supplementary Materials

The following supporting information can be downloaded at: https://www.mdpi.com/article/10.3390/children13081016/s1, Supplementary Material File S1: Original Italian version of the survey questionnaire.

## Abbreviations

The following abbreviations are used in this manuscript:

Abbreviation	Definition
FMT	Fecal Microbiota Transplantation
IQR	Interquartile Range
SD	Standard Deviation
NICU	Neonatal Intensive Care Unit
KAP	Knowledge, Attitudes, and Practices
GdS NeoPed MICS	Neonatal and Pediatric Clinical Microbiomics Study Group
