# Supplementary material for: Bridging Microbiota Science and Clinical Practice in Pediatric Healthcare: A National Survey
Source: Children (Basel). 2026 Jul 30;13(8):1016. doi: 10.3390/children13081016 (PMC13511371; doi:10.3390/children13081016)
Supplement: Supplementary file 1 [file children-13-01016-s001.zip › children-4423086-supplementary.pdf]

Supplementary File S1

English Version of the Original Questionnaire

SURVEY

Current Knowledge and Perceptions of the Role of the Microbiota in Child Development and Health: A Cross-Sectional Survey Among Pediatric Specialists

* Indicates a required question

Supplementary File S1

English Version of the Original Questionnaire

Dear Colleague,

This survey, promoted by GdSNeoPed.MICS (Study Group on Neonatal and Pediatric Clinical Microbiomics of the Microbiota International Clinical Society), aims to explore the level of perception, knowledge, and clinical use of the potential of the gut microbiota in the field of pediatric health.

The objective is to collect data from specialists and professionals from different clinical disciplines in pediatrics in order to map educational needs, clinical experience, and opportunities for personalized microbiomics applications during the first 1,000 days and beyond. Your anonymous participation (estimated time: 10 minutes) will contribute to the development of research projects and evidence-based interdisciplinary educational strategies.

If you have already received and completed this same survey previously, please do not complete it a second time.

Thank you for your contribution!

Informed Consent for Participation and Personal Data Processing

Dear Participant,

The data collected will be used exclusively for scientific research and educational dissemination purposes, in full compliance with EU Regulation 2016/679 (GDPR) on the protection of personal data. No identifying data will be collected, except for an email address that may be provided voluntarily at the end of the questionnaire, which will be used exclusively to provide updates on this research.

You have the right, at any time, to withdraw from completing the questionnaire and to request access to, modification, or deletion of your data, where identifiable, by contacting

the project coordinators at the following email address: gdsneoped.mics@yahoo.com. By proceeding with the questionnaire, you declare that you have understood the information provided above and consent to the processing of your data for the purposes indicated.

Supplementary File S1

English Version of the Original Questionnaire

Section 1 – Professional Profile

* I consent to participate in the study and to the processing of my data as described

○ I consent

○ I do not consent

What is your specialist/professional position? *

○ Community specialist/professional

○ Hospital specialist/professional

○ University specialist/professional

○ Specialist in training

Supplementary File S1

English Version of the Original Questionnaire

What is your clinical specialty? *

○ Pediatrician

○ Neonatologist

○ Midwife

○ Pediatric Surgeon

○ Pediatric Gastroenterologist

○ Pediatric Otolaryngologist

○ Pediatric Allergist

○ Pediatric Pulmonologist

○ Pediatric Endocrinologist

○ Pediatric Oncologist

○ Child Neuropsychiatrist

○ Dental Hygienist

○ Dentist

○ Other:

How many years have you been working in your field? *

○ I am not yet working

○ < 5 years

○ 5–10 years

○ 11–20 years

○ > 20 years

Supplementary File S1

English Version of the Original Questionnaire

Section 2 – Knowledge and Perceptions of the Microbiota

Where do you practice your profession/training (please specify region and province)? *

Your answer

Have you received training on the microbiota? *

○ Yes, through university education

○ Yes, I attended in-person courses

○ Yes, I attended online courses

○ Yes, through conferences

○ Yes, through the scientific literature

○ No

○ Other:

Using a scale from 1 (no knowledge), 2 (basic knowledge), 3 (intermediate knowledge), 4 (advanced knowledge), 5 (expert), how competent do you consider yourself regarding the following topics?

1 2 3 4 5

What is the microbiota? *

1 2 3 4 5

What are its functions? *

1 2 3 4 5

How is it structured? *

1 2 3 4 5

What interferes with its proper development? *

1 2 3 4 5

How can its modulation be influenced? *

1 2 3 4 5

How is it studied? *

1 2 3 4 5

Using a scale from 1 (no role), 2 (marginal role), 3 (intermediate role), 4 (significant role), 5 (determinant role), how strongly do you think the individual's microbiota is associated with the following clinical scenarios?

1 2 3 4 5

Interpretation of colon fecal microbiota test results in a clinical context *

1 2 3 4 5

Knowledge of the concepts of prebiotic, probiotic, synbiotic, and postbiotic *

1 2 3 4 5

Choosing the appropriate personalized microbiota-targeted therapeutic approach for the individual case (e.g., lifestyle, dietary habits, antibiotics, prebiotics, probiotics, postbiotics...) *

1 2 3 4 5

Neonatal sepsis/necrotizing enterocolitis/bronchopulmonary dysplasia of prematurity/retinopathy of prematurity/poor growth *

Supplementary File S1

English Version of the Original Questionnaire

Food allergies / wheezing / asthma / allergic rhinitis *

1 2 3 4 5

Vaccine response *

1 2 3 4 5

Susceptibility to recurrent infections *

1 2 3 4 5

Functional gastrointestinal disorders *

1 2 3 4 5

Dental caries / periodontal diseases *

1 2 3 4 5

Supplementary File S1

English Version of the Original Questionnaire

IBD *

1 2 3 4 5

Obesity / metabolic syndrome *

1 2 3 4 5

Autoimmune diseases *

1 2 3 4 5

Neurodevelopmental disorders (ASD, ADHD, schizophrenia, cognitive/behavioral disorders) *

1 2 3 4 5

Anxiety and depression *

1 2 3 4 5

Epilepsy *

1 2 3 4 5

Sleep disorders *

1 2 3 4 5

Atopic dermatitis, acne *

1 2 3 4 5

Pediatric oncological diseases (ALL, brain tumors, lymphomas) *

1 2 3 4 5

Therapeutic response and modulation of side effects in pediatric oncological diseases *

1 2 3 4 5

Supplementary File S1

English Version of the Original Questionnaire

Section 3 – Clinical and Therapeutic Use

Do you ever recommend interventions to modulate the microbiota for preventive purposes in your patients? *

○ Yes, often (more than once a week)

○ Sometimes (no more than once a month)

○ Rarely (less than 10 times a year)

○ Never

If the previous answer was positive, in which situation(s) do you recommend targeted microbiota interventions for preventive purposes? (multiple answers possible)

☐ Prevention of neonatal dysbiosis (born to: dysbiotic mother, preterm birth, cesarean section, formula feeding, intrapartum and/or early antibiotic exposure)

☐ Prevention of drug-induced dysbiosis (antibiotics, PPIs, neurotropic drugs, hormones...)

☐ To promote vaccine response

☐ In the presence of risk factors for atopic disease

☐ In the presence of risk factors for obesity and/or metabolic syndrome

☐ In the presence of risk factors for autoimmune disease

☐ Before travel to tropical areas

☐ Other:

Supplementary File S1

English Version of the Original Questionnaire

Do you ever recommend interventions to modulate the microbiota in patients with chronic or complex diseases? *

○ Yes, often (more than once a week)

○ Sometimes (no more than once a month)

○ Rarely (less than 10 times a year)

○ Never

If the previous answer was positive, in which situation(s) do you recommend targeted microbiota interventions? *

☐ Recurrent upper respiratory tract infections

☐ Recurrent urinary tract infections

☐ Functional gastrointestinal disorders

☐ IBD

☐ Oral diseases

☐ Obesity and metabolic syndrome

☐ Autoimmune diseases

☐ Atopic diseases

☐ Neuropsychiatric disorders

☐ Oncological diseases

☐ Other:

Supplementary File S1

English Version of the Original Questionnaire

Which tool(s) do you use or would you recommend to modulate the microbiota? *

☐ Lifestyle interventions

☐ Personalized nutrition

☐ Probiotics

☐ Prebiotics

☐ Synbiotics

☐ Postbiotics

☐ Antibiotics

☐ Herbal products

☐ Fecal microbiota transplantation

☐ None

Have you ever prescribed/recommended or received the report of a fecal microbiota analysis? *

○ Yes, often (more than once a week)

○ Sometimes (no more than once a month)

○ Rarely (less than 10 times a year)

○ Never

○ I am not familiar with this type of analysis

○ Patients are referred to me with a microbiota analysis already prescribed by another specialist

○ Patients are referred to me with a microbiota analysis already performed on their own initiative

Supplementary File S1

English Version of the Original Questionnaire

If you selected "Patients are referred to me with a microbiota analysis already prescribed by another specialist", please specify which specialist.

Your answer

If you do not prescribe microbiota analysis, or prescribe it only rarely, what are the reasons?

☐ Because of the cost of the test

☐ Because I would not know how to interpret it

☐ Because I would not know who to refer it to for interpretation

☐ Because I never encounter patients who would need it

If you prescribe microbiota analyses in your clinical practice, which type do you use?

○ I mainly prescribe 16S rRNA analysis

○ I mainly prescribe shotgun metagenomic analysis

If you prescribe fecal colonic microbiota tests mainly for specific clinical conditions, please specify which.

Your answer

Supplementary File S1

English Version of the Original Questionnaire

Section 4 – Educational Needs and Interests

If you prescribe or interpret fecal microbiota analyses, which clinical metadata do you request? *

☐ Family history

☐ Maternal obstetric and neonatal history (maternal diet/medications/antibiotics/toxic exposures during pregnancy and breastfeeding, pregnancy course, gestational age at birth, mode of delivery, intrapartum and early postnatal antibiotics)

☐ Physiological history (growth, psychomotor development, sleep quality, Bristol Stool Scale, gastrointestinal symptoms, breastfeeding, complementary feeding and dietary habits, lifestyle, exposure to pollutants/toxic substances/stress)

☐ Past medical history

☐ Recent medical history

☐ Vaccination history

☐ Medication history since birth

☐ Dietary history since birth

☐ Food diary

☐ Blood tests (please specify)

☐ Instrumental investigations (please specify)

☐ None

☐ Other:

If you answered "Blood tests" and/or "Instrumental investigations", please specify:

Your answer

Supplementary File S1

English Version of the Original Questionnaire

Would you like to learn more about the relationship between the microbiota and child health? *

○ Yes

○ No

○ Maybe

If the previous answer was positive, which topic(s) would interest you the most?

☐ Maternal microbiota and offspring microbiota development

☐ Microbiota and the immune system

☐ Microbiota and neurodevelopment

☐ Microbiota and axes

☐ Fecal microbiota analysis and its clinical interpretation

☐ Functional foods, dietary patterns, and lifestyle in microbiota modulation

☐ Clinical use of prebiotics/probiotics/postbiotics

☐ Other:

Would you be interested in participating in a study group or community of practice and/or research on these topics? *

○ Yes

○ No

○ Maybe

THANK YOU!

Thank you for participating! If you would like to receive updates on the survey results or future initiatives, you may leave your email address (optional).

Your answer

Supplementary File S1

English Version of the Original Questionnaire

Project Coordinator

Dr. Mariarosaria Matera

Project Lead, GdSNeoPed.MICS

gdsneoped.mics@yahoo.com

MICS Council Member

Dr. Valentina Biagioli

info@mics.ist

GdS Members

Andrea Manzi, Pediatrician (Pozzuoli); Giuseppe Varrasi, Pediatrician (Brescia); Ilaria Cavecchia, Infectious Diseases (Turin); Laura Pennazzi, Midwife and Nutrition Biologist (Rome); Sebastian Lugli, Dietitian and Nutritionist (Modena); Maria Teresa Illiceto, Pediatric Surgeon and Pediatric Gastroenterologist (Pescara); Martina Meocci, Pulmonologist (Siena); Vera Gandini, Pediatrician (Varese); Valentina Biagioli, Nutrition Biologist, PhD Candidate (Genoa); Mariarosaria Matera, Pediatrician and Neonatologist (Grosseto).
